# Supplementary material for: Consensus Recommendations for Hyperpolarized [1-13C]pyruvate MRI Multi-center Human Studies
Source: ArXiv. 2025 Apr 29:arXiv:2504.20440v1. Preprint. [Version 1] (PMC12060990)
Supplement: Supplement 1 [file NIHPP2504.20440v1-supplement-1.pdf]

Supplementary material for “Consensus Meeting Recommendations for Hyperpolarized [1-^{13}C]pyruvate MRI Multi-center Human Studies”

The supplementary material includes additional details from the consensus process such as all statements posed to the members, the detailed results of scoring, and information about the participants.

Tables:

1. Information about all group members who participated.
2. Listing of all statements along with a summary of round 2 scoring indicating the most common outcome (Disagreement/Uncertain/Agreement) and whether consensus was reached (Consensus/No consensus).
3. Summary of all statements scoring with outcomes and consensus for round 1 and round 2.
4. Statements altered between round 1 and round 2.

*Supplementary Table 1: Group Members participating in the consensus voting (name, speciality, country/site, years of C13 expertise). Panellist with * contributed to round 1 scoring but were not able to participate in round 2 face-to-face meeting.*

Name	Specialty	Country/site	Years of expertise
Esben Sovso Szocska	Engineering / Physics	Aarhus University	10
Chris Laustsen	Engineering / Physics, Pharmacy Production & Manufacturing	Aarhus University	15
Lotte Bonde Bertelsen	Engineering / Physics, Pharmacy Production & Manufacturing	Aarhus University	10
Ferdia Gallagher	Clinician	Cambridge University	18
Ashley Grimmer	Pharmacy Production & Manufacturing	Cambridge University	6.5
Mary McLean	Engineering / Physics	Cambridge University	10
Ching-Yi Hsieh	Engineering / Physics	Chang Gung University and Chang Gung Memorial Hospital at Linkou, Taiwan	6

Sebastian Kozerke *	Engineering / Physics	ETH Zurich	15
Albert Chen *	Engineering / Physics	GE Healthcare	20
Adam Gaunt	Engineering / Physics, Other	GE Healthcare	10
Arnaud Comment	Engineering / Physics, Pharmacy Production & Manufacturing	GE Healthcare	15
Jim Bankson	Engineering / Physics	MD Anderson Cancer Center	14
Kayvan Keshari	Engineering / Physics	Memorial Sloan Kettering - Cancer Center	15
James Grist	Engineering / Physics	Nottingham University & Oxford University	10
Damian Tyler	Engineering / Physics	Oxford University	19
Fulvio Zaccagna	Radiologist / Radiographer, Clinician	Oxford University & Cambridge University	9

Titus Lanz *	Engineering / Physics	RAPID Biomedical GmbH	20
Jan Henrik Ardenkjær-Larsen	Engineering / Physics	Technical University of Denmark	25
Mathilde Lerche	Engineering / Physics, Other	Technical University of Denmark	22
Peder Larson	Engineering / Physics	UCSF	17
Duan Xu	Engineering / Physics	UCSF	13
Bob Bok	Clinician	UCSF	17
Jim Slater	Pharmacy Production & Manufacturing	UCSF	8
Duy Dang	Pharmacy Production & Manufacturing	UCSF	2
Adam Autry	Engineering / Physics	UCSF	8
Rafat Chowdhury	Engineering / Physics	University College London	7
Richard Hesketh	Radiologist / Radiographer, Clinician	University College London	6

Shonit Punwani	Engineering / Physics, Radiologist / Radiographer	University College London	9
Dirk Mayer	Engineering / Physics	University of Maryland	17
Chuck Cunningham	Engineering / Physics	University of Toronto	20
Jae Mo Park	Engineering / Physics	UT Southwestern	14
Vlad Zaha	Radiologist / Radiographer, Clinician	UT Southwestern	6

Supplementary Table 2: Summary of round 2 scoring for all statements included. The outcome category (1-3 disagree, 4-6 uncertain or 7-9 agree) was defined by the median score across all non-zero responders. Consensus indicates whether a 66% or more of the non-zero responses were in the outcome category when at least 14 votes were made.

No.	Statement	Outcome (round 2)	Consensus (round 2)	Fraction	Percen tage
**1A-1: For multi-center studies:**					

**1**	a. all sites have to follow the same 13C pyruvate preparation guidelines (e.g. Sterile Compounding or Terminal Sterilization).	Disagreement	Consensus	17/25	68%
**2**	b. it is preferred that all sites follow the same 13C pyruvate preparation guidelines.	Agreement	No consensus	15/25	60%
**1A-2: For multi-center studies:**					
**3**	a. all sites have to follow the same SOPs for 13C pyruvate preparation process.	Disagreement	Consensus	20/26	77%
**4**	b. it is preferred that all sites follow the same SOPs for 13C pyruvate preparation process.	Agreement	No consensus	15/26	58%
**1A-3: For multi-center studies:**					
**5**	a. all sites have to have the same environment and facilities (e.g. clean room, isolator, clean bench) for 13C pyruvate preparation process.	Disagreement	Consensus	19/26	73%
**6**	b. it is preferred that all sites have the same environment and facilities for 13C pyruvate preparation process.	Agreement	No consensus	13/26	50%
**1B-1: For multi-center studies:**					
**7**	a. all sites have to follow the same release criteria for release of hyperpolarized 13C pyruvate.	Agreement	Consensus	21/26	81%
**8**	b. it is preferred that all sites follow the same release criteria for release of hyperpolarized 13C pyruvate.	Agreement	Consensus	25/26	96%
**1B-2: The following have to be part of the dose release criteria:**					

**9**	a. PA concentration	Agreement	Consensus	25/26	96%
**10**	b. EPA concentration	Agreement	Consensus	26/26	100%
**11**	c. pH	Agreement	Consensus	26/26	100%
**12**	d. Temperature	Agreement	Consensus	23/26	88%
**13**	e. Volume	Agreement	Consensus	21/26	81%
**14**	f. Polarization	Agreement	No consensus	14/25	56%
**1B-3: For multi-center studies:**					
**15**	a. all sites have to use the same QC system measurement for dose release.	Disagreement	Consensus	17/24	71%
**16**	b. it is preferred that all sites use the same QC system measurement for dose release.	Uncertain	No consensus	7/26	27%
**2A-1: For multi-center studies:**					
**17**	a. all sites have to use a scanner from the same manufacturer. (If you are a direct employee of any of the manufacturers listed below, please select “0”)	Disagreement	Consensus	19/22	86%
**18**	b. it is preferred that all sites use a scanner from the same manufacturer.	Disagreement	Consensus	17/23	74%

**2A-2: Viable options for participation in multi-center studies are: (If you are a direct employee of any of the manufacturers listed below, please select "0")**					
**19**	a. GE	Agreement	Consensus	22/22	100%
**20**	b. Siemens	Agreement	Consensus	21/22	95%
**21**	c. Phillips	Agreement	Consensus	17/19	89%
**2A-3: Preferred manufacturer is: (If you are a direct employee of any of the manufacturers listed below, please select "0")**					
**22**	a. GE	Agreement	No consensus	13/20	65%
**23**	b. Siemens	Uncertain	No consensus	10/20	50%
**24**	c. Phillips	Uncertain	No consensus	10/19	53%
**2A-4: For multi-center studies:**					
**25**	a. the magnet field strength B0 has to be the same for all sites.	Agreement	Consensus	19/24	79%
**26**	b. it is preferred that all sites use a scanner with the same B0.	Agreement	Consensus	23/24	96%
**2A-5: Viable options for participation in multi-center study are:**		*Question was removed*			
**27**	a. 0.5T				

**28**	b. 1.5T				
**29**	c. 3T				
**30**	d. 7T				
**2A-6: Preferred B0 is:**					
**31**	a. 0.5T	Disagreement	Consensus	19/23	83%
**32**	b. 1.5T	Uncertain	Consensus	16/24	67%
**33**	c. 3T	Agreement	Consensus	23/24	96%
**34**	d. 7T	Uncertain	No consensus	11/24	46%
**2A-7: For multi-center studies:**					
**35**	a. all sites have to have the same maximum gradient strength.	Agreement	No consensus	13/23	57%
**36**	b. it is preferred that all sites have the same maximum gradient strength.	Agreement	Consensus	22/23	96%
**2A-8: For multi-center studies:**					
**37**	a. all sites have to have the same maximum gradient slew rate.	Agreement	No consensus	14/23	61%

**38**	b. it is preferred that all sites have the same maximum gradient slew rate.	Agreement	Consensus	22/23	96%
**2A-9: For multi-center studies:**					
**39**	a. all sites have to have the same maximum available RF amplifier power.	Agreement	No consensus	12/23	52%
**40**	b. it is preferred that all sites have the same maximum available amplifier power.	Agreement	Consensus	22/23	96%
**2A-10: For multi-center studies:**					
**41**	a. all sites have to have the same manufacturer and model for 13C transmit and receive coils.	Disagreement	No consensus	13/24	54%
**42**	b. it is preferred that all sites have the same manufacturer and model for 13C transmit and receive coils.	Agreement	Consensus	20/24	83%
**2A-11: For multi-center studies:**					
**43**	a. all sites have to have the same fundamental geometry design for 13C transmit and receive coils.	Uncertain	No consensus	6/23	26%
**44**	b. it is preferred that all sites have the same fundamental geometry design for 13C transmit and receive coils.	Agreement	Consensus	22/23	96%
**2A-12: For multi-center studies:**					
**45**	a. all sites have to have a reference phantom in the field of view of 13C coil.	Uncertain	No consensus	8/24	33%
**46**	b. it is preferred that all sites have to have a reference phantom in the field of view of 13C coil.	Agreement	No consensus	15/24	63%

**2A-13: For multi-center studies:**					
**47**	a. all sites have to be able to acquire 1H and 13C images without repositioning the patient in between acquisition scans.	Agreement	Consensus	16/24	67%
**48**	b. it is preferred that all sites are able to acquire 1H and 13C images without repositioning the patient in between acquisition scans.	Agreement	Consensus	23/24	96%
**2B-1: For multi-center studies:**					
**49**	a. all sites have to perform an agreed-upon scanner site qualification (e.g. QC experiments).	Agreement	Consensus	23/24	96%
**50**	b. it is preferred that all sites perform an agreed-upon scanner site qualification.	Agreement	Consensus	24/24	100%
**2B-2: Viable approaches to scanner site qualification include use of:**					
**51**	a. Static thermal phantoms	Agreement	Consensus	25/25	100%
**52**	b. Dynamic HP phantoms (e.g. with enzymatic conversion)	Uncertain	No consensus	5/25	20%
**53**	c. Travelling humans injected with HP 13C	Uncertain	No consensus	8/24	33%
**2B-3: Preferred approach is:**					
**54**	a. Static thermal phantoms	Agreement	Consensus	25/25	100%

**55**	b. Dynamic HP phantoms (e.g. with enzymatic conversion)	Uncertain	No consensus	11/25	44%
**56**	c. Travelling humans injected with HP 13C	Disagreement	No consensus	12/24	50%
**2B-4: For multi-center studies:**					
**57**	a. all sites have to perform on-going hardware quality assurance throughout the study.	Agreement	Consensus	20/25	80%
**58**	b. it is preferred that all sites perform on-going hardware quality assurance throughout the study.	Agreement	Consensus	24/25	96%
**2C-1: For multi-center studies:**					
**59**	a. all sites have to follow the same protocol for adjustment of RF pulse power.	Disagreement	No consensus	11/22	50%
**60**	b. it is preferred that all sites follow the same protocol for adjustment of RF pulse power.	Agreement	Consensus	17/22	77%
**2C-2: Viable options for pre-scan setting of RF pulse power are:**					
**61**	a. Automated optimization, e.g. Bloch-Siegert	Agreement	Consensus	21/22	95%
**62**	b. Manual optimization, e.g. power sweep	Agreement	Consensus	22/22	100%
**63**	c. Predetermined values from phantom calibrations	Agreement	No consensus	13/22	59%
**2C-3: Preferred method of adjustment of RF pulse power:**					

**64**	a. Automated optimization, e.g. Bloch-Siegert	Agreement	Consensus	21/21	100%
**65**	b. Manual optimization, e.g. power sweep	Agreement	Consensus	19/21	90%
**66**	c. Predetermined values from phantom calibrations	Uncertain	No consensus	7/21	33%
**2C-4: For multi-center studies:**					
**67**	a. all sites have to follow the same protocol for pre-scan adjustment of center frequency.	Uncertain	No consensus	4/24	17%
**68**	b. it is preferred that all sites follow the same protocol for pre-scan adjustment of center frequency.	Agreement	Consensus	23/24	96%
**2C-5: Viable options for pre-scan setting of center frequency are:**					
**69**	a. 1H frequency in tissue	Agreement	Consensus	24/24	100%
**70**	b. 13C frequency in phantom	Agreement	Consensus	17/24	71%
**71**	c. 13C frequency in tissue	Uncertain	No consensus	11/24	46%
**72**	d. 23Na frequency in tissue	Uncertain	No consensus	12/24	50%
**2C-6: Preferred method of adjustment of center frequency is:**					
**73**	a. 1H frequency in tissue	Agreement	Consensus	23/24	96%

**74**	b. 13C frequency in phantom	Uncertain	No consensus	9/24	38%
**75**	c. 13C frequency in tissue	Uncertain	No consensus	10/24	42%
**76**	d. 23Na frequency in tissue	Uncertain	No consensus	11/24	46%
**2C-7: For multi-center studies:**					
**77**	a. all sites have to follow the same protocol for adjustment of receive gains.	Uncertain	No consensus	7/21	33%
**78**	b. it is preferred that all sites follow the same protocol for adjustment of receive gains.	Uncertain	No consensus	6/21	29%
**2C-8: Viable options for pre-scan setting of receive gains are:**					
**79**	a. Maximum values possible	Disagreement	No consensus	11/21	52%
**80**	b. Based on previous in vivo measurements	Agreement	Consensus	19/22	86%
**81**	c. Based on phantom measurements	Agreement	No consensus	12/22	55%
**2C-9: Preferred method of adjustment of receive gains is:**					
**82**	a. Maximum values possible	Disagreement	No consensus	11/21	52%
**83**	b. Based on previous in vivo measurements	Agreement	Consensus	19/22	86%

**84**	c. Based on phantom measurements	Agreement	No consensus	12/22	55%
**2C-10: For multi-center studies:**					
**85**	a. all sites have to follow the same protocol for shimming to optimize B0 homogeneity.	Uncertain	No consensus	7/24	29%
**86**	b. it is preferred that all sites follow the same protocol for shimming to optimize B0 homogeneity.	Agreement	Consensus	21/24	88%
**2C-11: Viable options for shimming are:**					
**87**	a. Automated shimming over imaging volume	Agreement	Consensus	22/23	96%
**88**	b. Automated shimming over a ROI (e.g. PRESS Box)	Agreement	Consensus	21/23	91%
**89**	c. Manual shimming over imaging volume	Uncertain	No consensus	6/23	26%
**90**	d. Manual shimming over a ROI	Agreement	No consensus	12/23	52%
**2C-12: Preferred methods of adjustment of shims are:**					
**91**	a. Automated shimming over imaging volume	Agreement	Consensus	22/23	96%
**92**	b. Automated shimming over a ROI (e.g. PRESS Box)	Agreement	Consensus	22/23	96%
**93**	c. Manual shimming over imaging volume	Uncertain	No consensus	11/23	48%

**94**	d. Manual shimming over a ROI	Uncertain	No consensus	10/23	43%
**2C-13: For multi-center studies:**					
**95**	a. all sites have to perform higher-order shimming.	Disagreement	No consensus	15/24	63%
**96**	b. it is preferred that all sites perform higher-order shimming.	Uncertain	No consensus	6/24	25%
**3A-1: For multi-center studies:**					
**97**	a. all sites have to use the same pulse sequence type (e.g. MRS/I, metabolite-specific imaging, or chemical shift encoding) when aggregating data across sites.	Uncertain	No consensus	10/26	38%
**98**	b. it is preferred that all sites use the same pulse sequence type when aggregating data across sites.	Agreement	Consensus	25/26	96%
**3A-2: For multi-center studies:**					
**99**	a. all sites have to use sequences that provide spatial localization.	Agreement	No consensus	14/26	54%
**100**	b. it is preferred that all sites use sequences that provide spatial localization.	Agreement	Consensus	22/26	85%
**3A-3: For multi-center studies:**					
**101**	a. all sites have to use sequences that provide spectral information (e.g. generate metabolite maps, provide a spectrum).	Uncertain	No consensus	9/26	35%

**102**	b. it is preferred that all sites use sequences that provide spectral information (e.g. generate metabolite maps, provide a spectrum).	Agreement	Consensus	26/26	100%
**3A-4: For multi-center studies:**					
**103**	a. all sites have to use sequences that provide dynamic (time resolved) data.	Uncertain	No consensus	8/26	31%
**104**	b. it is preferred that all sites use sequences that provide dynamic (time resolved) data.	Agreement	Consensus	24/26	92%
**3A-5: For multi-center studies:**					
**105**	a. all sites have to capture the bolus of pyruvate in the data acquisition.	Uncertain	No consensus	11/26	42%
**106**	b. it is preferred that all sites capture the bolus of pyruvate in the data acquisition.	Agreement	Consensus	23/26	88%
**3A-6: For a given pulse sequence:**					
**107**	a. all sites have to use the same flip angle scheme.	Uncertain	No consensus	4/26	15%
**108**	b. it is preferred that all sites use the same flip angle scheme.	Agreement	Consensus	25/26	96%
**3A-7: For multi-center studies:**					
**109**	a. all sites have to use the same spatial resolution when aggregating data across sites.	Uncertain	No consensus	9/26	35%

**110**	b. it is preferred that all sites use the same spatial resolution when aggregating data across sites.	Agreement	Consensus	25/26	96%
**3A-8: For multi-center studies:**					
**111**	a. all sites have to have the same timing parameters (start time, temporal resolution, and number of timeframes) when aggregating data across different sites.	Uncertain	No consensus	7/26	27%
**112**	b. it is preferred that all sites have the same timing parameters when aggregating data across different sites.	Agreement	Consensus	25/26	96%
**3A-9: For multi-center studies:**					
**113**	a. all sites have to use the same undersampling method (e.g. partial Fourier acceleration, parallel imaging acceleration, model-based acceleration methods such as compressed sensing, low-rank reconstructions, and deep learning).	Uncertain	No consensus	4/23	17%
**114**	b. it is preferred that all sites have the same undersampling method (e.g. partial Fourier acceleration, parallel imaging acceleration, model-based acceleration methods such as compressed sensing, low-rank reconstructions, and deep learning).	Agreement	Consensus	22/23	96%
**3A-10: Best practice recommendation**					
**115**	a. Any contrast injection needed for 1H MRI have to be performed after the HP 13C study.	Agreement	Consensus	24/25	96%
**116**	b. 1H images with coverage equal to or exceeding the 13C FOV have to be acquired, for anatomic reference.	Agreement	Consensus	26/26	100%

**117**	c. Acquire a B0 field map to identify and potentially correct for artifacts caused by B0 inhomogeneity	Agreement	Consensus	25/26	96%
**3B-1: Best practice recommendation for PROSTATE acquisition includes:**					
**118**	a. Multi-shot readouts	Uncertain	No consensus	7/15	47%
**119**	b. Single-shot readouts	Agreement	No consensus	8/15	53%
**120**	c. Metabolite-specific RF excitation	Agreement	Consensus	13/15	87%
**121**	d. Resolve a spectrum	Agreement	Consensus	13/15	87%
**122**	e. Respiratory gating	Disagreement	Consensus	13/15	87%
**123**	f. Cardiac gating	Disagreement	Consensus	14/15	93%
**3B-2: Best practice recommendation for BRAIN acquisition includes:**					
**124**	a. Multi-shot readouts	Uncertain	No consensus	7/19	37%
**125**	b. Single-shot readouts	Agreement	Consensus	14/19	74%
**126**	c. Metabolite-specific RF excitation	Agreement	Consensus	17/19	89%
**127**	d. Resolve a spectrum	Agreement	Consensus	14/19	74%

**128**	e. Respiratory gating	Disagreement	Consensus	18/20	90%
**129**	f. Cardiac gating	Disagreement	Consensus	18/20	90%
**3B-3: Best practice recommendation for CARDIAC acquisition includes:**					
**130**	a. Multi-shot readouts	Disagreement	No consensus	8/14	57%
**131**	b. Single-shot readouts	Agreement	Consensus	12/14	86%
**132**	c. Metabolite-specific RF excitation	Agreement	Consensus	12/14	86%
**133**	d. Resolve a spectrum	Agreement	Consensus	10/14	71%
**134**	e. Respiratory gating	Agreement	Consensus	13/15	87%
**135**	f. Cardiac gating	Agreement	Consensus	14/15	93%
**3B-4: Best practice recommendation for ABDOMEN acquisition includes:**					
**136**	a. Multi-shot readouts	Uncertain	No consensus	10/19	53%
**137**	b. Single-shot readouts	Agreement	Consensus	13/19	68%
**138**	c. Metabolite-specific RF excitation	Agreement	Consensus	17/20	85%

**139**	d. Resolve a spectrum	Agreement	Consensus	15/20	75%
**140**	e. Respiratory gating	Agreement	Consensus	18/20	90%
**141**	f. Cardiac gating	Uncertain	No consensus	8/20	40%
**3C-1: For multi-center studies:**					
**142**	a. a standardized reconstruction pipeline has to be used when aggregating data.	Agreement	No consensus	14/25	56%
**143**	b. it is preferred that a standardized reconstruction pipeline is used when aggregating data.	Agreement	Consensus	25/25	100%
**3C-2: Best practice recommendation includes:**					
**144**	a. Using denoising techniques to denoise 13C data.	Uncertain	No consensus	11/24	46%
**145**	b. Sum-of-squares combination of multi-channel data.	Uncertain	No consensus	4/20	20%
**146**	c. HP data-driven approaches for combination of multi-channel data.	Agreement	No consensus	13/20	65%
**147**	d. Zero-filling prior to analysis.	Agreement	No consensus	10/20	50%
**148**	e. Spatial filtering prior to analysis.	Uncertain	No consensus	8/21	38%
**149**	f. Spectral filtering prior to analysis.	Agreement	No consensus	12/21	57%

**150**	g. Spatial distortion correction (e.g. EPI displacement and ghosting artifact corrections, spiral off-resonance correction).	Agreement	Consensus	22/23	96%
**3D-1: To facilitate analysis, all sites in a multi-center study have to record the following study parameters:**					
**151**	a. ‘Standard’ scan parameters, such as TR, TE, FOV, matrix size, and bandwidth need to be reported by each site.	Agreement	Consensus	25/25	100%
**152**	b. Timing parameters for the pyruvate injection and flush (start, duration) and acquisition (start : interval : end) need to be reported by each site.	Agreement	Consensus	24/25	96%
**153**	c. If a spectrally-selective RF pulse is used, the frequency response specifications and frequency offsets for each metabolite need to be clearly stated by each site.	Agreement	Consensus	24/25	96%
**154**	d. If a variable flip angle scheme is used, the flip angles for each metabolite and each timepoint need to be reported by each site.	Agreement	Consensus	25/25	100%
**155**	e. If a multi-echo readout is used for chemical shift encoding, the echo-spacing, number of echoes, and metabolite frequencies used in the reconstruction should be reported by each site.	Agreement	Consensus	25/25	100%
**4A-1: For multi-center studies:**					
**156**	a. all sites have to store all 13C k-space raw data in addition to reconstructed images/spectra.	Agreement	No consensus	17/26	65%
**157**	b. it is preferred that all sites have to store all 13C k-space raw data in addition to reconstructed images/spectra.	Agreement	Consensus	26/26	100%
**4A-2: Viable data for storage includes:**					
**158**	a. Raw data (k-space)	Agreement	Consensus	25/25	100%

**159**	b. Minimally processed data (reconstructed images/spectra)	Agreement	Consensus	25/25	100%
**160**	c. Processed data (parameter maps)	Agreement	Consensus	24/25	96%
**4A-3: Preferred data for storage are:**					
**161**	a. Raw data (k-space)	Agreement	Consensus	25/25	100%
**162**	b. Minimally processed data (reconstructed images/spectra)	Agreement	Consensus	24/25	96%
**163**	c. Processed data (parameter maps)	Agreement	Consensus	23/25	92%
**4A-4: For multi-center studies, the following additional data has to be recorded:**					
**164**	a. QC - pyruvate concentration	Agreement	Consensus	25/28	89%
**165**	b. QC - EPA concentration	Agreement	Consensus	23/28	82%
**166**	c. QC - Polarization	Agreement	No consensus	17/28	61%
**167**	d. QC - pH	Agreement	Consensus	24/28	86%
**168**	e. QC - temperature	Agreement	Consensus	21/28	75%
**169**	f. Volume injected	Agreement	Consensus	26/28	93%

**170**	g. Injection rate	Agreement	Consensus	25/28	89%
**171**	h. Elapsed time from dissolution to start of injection	Agreement	Consensus	23/28	82%
**172**	i. Elapsed time between start of injection and start of data acquisition	Agreement	Consensus	24/28	86%
**4A-5: It is preferred that the following additional data are recorded:**					
**173**	a. QC - pyruvate concentration	Agreement	Consensus	27/28	96%
**174**	b. QC - EPA concentration	Agreement	Consensus	26/28	93%
**175**	c. QC - Polarization	Agreement	Consensus	26/28	93%
**176**	d. QC - pH	Agreement	Consensus	27/28	96%
**177**	e. QC - temperature	Agreement	Consensus	25/28	89%
**178**	f. Volume injected	Agreement	Consensus	27/28	96%
**179**	g. Injection rate	Agreement	Consensus	28/28	100%
**180**	h. Elapsed time from dissolution to start of injection	Agreement	Consensus	28/28	100%
**181**	i. Elapsed time between start of injection and start of data acquisition	Agreement	Consensus	28/28	100%

**4A-6: Basic patient metadata to be recorded/stored:**		*Question was removed*			
**182**	a. Diagnosis/disease				
**183**	b. Disease stage (e.g. TNM status, grade category)				
**184**	c. Age				
**185**	d. Gender				
**186**	e. Ethnicity				
**187**	f. Weight and Height				
**188**	g. Vital signs on day of study (e.g. blood pressure, heart rate)				
**4B-1: For multi-center studies:**					
**189**	a. data quality assessment has to be made.	Agreement	Consensus	25/26	96%
**190**	b. it is preferred that data quality assessment to be made.	Agreement	Consensus	25/26	96%
**4B-2: Viable method to assess data quality are:**					
**191**	a. SNR Measurements	Agreement	Consensus	26/26	100%

**192**	b. Assessment of artifact levels	Agreement	Consensus	22/26	85%
**193**	c. Assessment of dynamic signal curves	Agreement	Consensus	18/25	72%
**194**	d. External Reference-based assessments	Agreement	Consensus	21/25	84%
**4B-3: Preferred method to assess data quality are:**					
**195**	a. SNR Measurements	Agreement	Consensus	26/26	100%
**196**	b. Assessment of artifact levels	Agreement	Consensus	21/26	81%
**197**	c. Assessment of dynamic signal curves	Agreement	Consensus	21/25	84%
**198**	d. External Reference-based assessments	Agreement	No consensus	15/23	65%
**4B-4: Viable method for calculating 13C SNR for data quality assessment includes:**					
**199**	a. Temporal peak Pyruvate SNR	Agreement	Consensus	23/24	96%
**200**	b. Temporal AUC Pyruvate SNR	Agreement	Consensus	23/24	96%
**201**	c. Temporal peak total Carbon SNR	Agreement	Consensus	23/24	96%
**202**	d. Temporal AUC total Carbon SNR	Agreement	Consensus	23/24	96%

**4B-5: Preferred method for calculating 13C SNR for data quality assessment are:**					
**203**	a. Temporal peak Pyruvate SNR	Agreement	No consensus	15/24	63%
**204**	4b. Temporal AUC Pyruvate SNR	Agreement	Consensus	19/24	79%
**205**	c. Temporal peak total Carbon SNR	Agreement	Consensus	18/24	75%
**206**	d. Temporal AUC total Carbon SNR	Agreement	Consensus	22/24	92%
**4C-1: For multi-center studies:**					
**207**	a. HP MRI data has to be evaluated with quantitative metabolism metrics.	Agreement	No consensus	13/26	50%
**208**	b. it is preferred that HP MRI data to be evaluated with quantitative metabolism metrics.	Agreement	Consensus	25/26	96%
**4C-2: Viable options for evaluating HP MRI data include calculation of:**					
**209**	a. Metabolite signals	Agreement	No consensus	16/25	64%
**210**	b. Normalized Metabolite signals	Agreement	Consensus	23/25	92%
**211**	c. Metabolite AUCs	Agreement	Consensus	21/25	84%
**212**	d. Normalized Metabolite AUCs	Agreement	Consensus	23/25	92%

**213**	e. Metabolite AUC ratios	Agreement	Consensus	25/25	100%
**214**	f. Kinetic rates	Agreement	Consensus	23/25	92%
**4C-3: The preferred metric for evaluating HP MRI data is:**					
**215**	a. Metabolite signals	Agreement	No consensus	13/26	50%
**216**	b. Normalized Metabolite signals	Agreement	Consensus	18/26	69%
**217**	c. Metabolite AUCs	Agreement	Consensus	17/25	68%
**218**	d. Normalized Metabolite AUCs	Agreement	Consensus	23/25	92%
**219**	e. Metabolite AUC ratios	Agreement	Consensus	24/25	96%
**220**	f. Kinetic rates	Agreement	Consensus	19/25	76%
**4C-4: Multi-center HP MRI studies:**					
**221**	a. have to incorporate a measurement of tissue perfusion to explore as a covariate with other metrics.	Uncertain	No consensus	7/25	28%
**222**	b. it is preferred that multi-center HP MRI studies incorporate a measurement of tissue perfusion to explore as a covariate with other metrics.	Agreement	Consensus	20/25	80%

4D-1: For multi-center studies:					
**223**	a. an assessment of repeatability has to be made.	Agreement	Consensus	24/26	92%
**224**	b. it is preferred that an assessment of repeatability to be made.	Agreement	Consensus	26/26	100%
**4D-2: Viable options for assessing repeatability include use of:**					
**225**	a. Static thermal phantoms	Agreement	Consensus	22/25	88%
**226**	b. Dynamic phantoms (e.g. enzymatic conversion)	Agreement	No consensus	16/25	64%
**227**	c. Same Subject injected with repeat HP 13C injections	Agreement	Consensus	21/25	84%
**4D-3: Preferred options for assessing repeatability include use of:**					
**228**	a. Static thermal phantoms	Agreement	Consensus	20/25	80%
**229**	b. Dynamic phantoms (e.g. enzymatic conversion)	Agreement	No consensus	14/25	56%
**230**	c. Same Subject injected with repeat HP 13C injections	Agreement	Consensus	21/25	84%
**4D-4: For multi-center studies:**					
**231**	a. an assessment of reproducibility has to be made.	Agreement	Consensus	21/25	84%

**232**	b. it is preferred that an assessment of reproducibility to be made.	Agreement	Consensus	25/25	100%
**4D-5: Viable options for assessing reproducibility include use of:**					
**233**	a. Static thermal phantoms	Agreement	Consensus	22/25	88%
**234**	b. Dynamic phantoms (e.g. enzymatic conversion)	Agreement	Consensus	18/25	72%
**235**	c. Same Subject injected with HP 13C injections at more than one site	Agreement	Consensus	20/25	80%
**4D-6: Preferred options for assessing reproducibility include use of:**					
**236**	a. Static thermal phantoms	Agreement	Consensus	18/25	72%
**237**	b. Dynamic phantoms (e.g. enzymatic conversion)	Agreement	No consensus	14/25	56%
**238**	c. Same Subject injected with HP 13C injections at more than one site	Agreement	Consensus	19/25	76%
**4E-1: Best practice for visualizing hyperpolarized MR data includes:**					
**239**	a. Anatomical reference image required	Agreement	Consensus	24/25	96%
**240**	b. Metabolite maps overlaid on anatomy	Agreement	Consensus	23/25	92%
**241**	c. Metabolite maps next to anatomy	Agreement	No consensus	16/25	64%

**242**	d. Images without masking to ROIs	Agreement	Consensus	19/25	76%
**243**	e. Use of transparency in overlaid maps	Agreement	Consensus	22/25	88%
**244**	f. Interpolation	Uncertain	No consensus	11/25	44%
**4E-2: For multi-center studies:**					
**245**	a. all sites have to use the same visualization methodology when evaluating data.	Agreement	Consensus	19/25	76%
**246**	b. it is preferred that all sites to use the same visualization methodology when evaluating data.	Agreement	Consensus	25/25	100%

Supplementary Table 3: Summary of how all statements scored by participants during Round 1 and Round 2.

**Consensus round**	**Agreement with consensus,** *n* (%)	**Disagreement with consensus,** *n* (%)	**Uncertainty or no consensus,** *n* (%)
Round 1 (*n*=246)	133 (54.1%)	3 (1.2%)	110 (44.7%)
Round 2 (*n*=235)	146 (62.1%)	11 (4.7%)	74 (33.2%)

Supplementary Table 4: Summary of statement additions, deletions, and changes made after round 1 and during round 2.

**Altered statement post- round 2**	**Previous statement**	**New statement**
	2A-5: Viable options for participation in multi-center study are:	Removed
**27**	a. 0.5T	Removed
**28**	b. 1.5T	Removed
**29**	c. 3T	Removed
**30**	d. 7T	Removed
	4A-4: For multi-center studies, the following additional data has to be **recorded**:	4A-4: For multi-center studies, the following additional data has to be **recorded and reported**:
**164**	a. QC - pyruvate concentration	a. QC - pyruvate concentration
**165**	b. QC - EPA concentration	b. QC - EPA concentration
**166**	c. QC - Polarization	c. QC - Polarization
**167**	d. QC - pH	d. QC - pH
**168**	e. QC - temperature	e. QC - temperature
**169**	f. Volume injected	f. Volume injected
**170**	g. Injection rate	g. Injection rate
**171**	h. Elapsed time from dissolution to start of injection	h. Elapsed time from dissolution to start of injection

**172**	i. Elapsed time between start of injection and start of data acquisition	i. Elapsed time between start of injection and start of data acquisition
	4A-5: It is preferred that the following additional data are **recorded**:	4A-5: It is preferred that the following additional data are **recorded and reported**:
**173**	a. QC - pyruvate concentration	a. QC - pyruvate concentration
**174**	b. QC - EPA concentration	b. QC - EPA concentration
**175**	c. QC - Polarization	c. QC - Polarization
**176**	d. QC - pH	d. QC - pH
**177**	e. QC - temperature	e. QC - temperature
**178**	f. Volume injected	f. Volume injected
**179**	g. Injection rate	g. Injection rate
**180**	h. Elapsed time from dissolution to start of injection	h. Elapsed time from dissolution to start of injection
**181**	i. Elapsed time between start of injection and start of data acquisition	i. Elapsed time between start of injection and start of data acquisition
	4A-6: Basic patient metadata to be recorded/stored:	Removed
**182**	a. Diagnosis/disease	Removed
**183**	b. Disease stage (e.g. TNM status, grade category)	Removed
**184**	c. Age	Removed
**185**	d. Gender	Removed
**186**	e. Ethnicity	Removed
**187**	f. Weight and Height	Removed

**188**	g. Vital signs on day of study (e.g. blood pressure, heart rate)	Removed
